# PLXNA3 is overexpressed and promotes cell proliferation and migration in colorectal cancer

**DOI:** 10.1007/s12672-026-05355-9

**Published:** 2026-05-31

**Authors:** Shun Ling, Zhijie Xiong, Dezhong Yan, Jiahui Feng

**Affiliations:** 1 Department of Gastroenterology, Loudi Central Hospital, Loudi, 417000 Hunan China; 2 Loudi Hospital, The First Affiliated Hospital of Guangzhou Medical University, Loudi, 417000 Hunan China

**Keywords:** PLXNA3, Colorectal cancer, Prognosis, Immune infiltration, Cell proliferation, Cell migration, Experimental validation

## Abstract

**Background:**

Plexins (PLXNs) are cell surface receptors for class 3 semaphorins and play critical roles in immune regulation and tumorigenesis. Plexin A3 (PLXNA3) is involved primarily in nervous system development, but its function in colorectal cancer (CRC) remains poorly understood. Recent computational studies have identified PLXNA3 as a potential prognostic biomarker in CRC, yet experimental validation of its biological role is lacking.

**Methods:**

PLXNA3 expression and its prognostic significance were analyzed using data from The Cancer Genome Atlas (TCGA) and Gene Expression Omnibus (GEO) databases. Immune infiltration correlations were assessed via TIMER2.0. Gene set enrichment analysis (GSEA) was performed to predict potential biological functions. For experimental validation, qRT‒PCR was used to assess PLXNA3 expression in CRC cell lines, and CCK-8, colony formation, and Transwell assays were conducted to evaluate the effects of PLXNA3 knockdown on cell proliferation and migration.

**Results:**

PLXNA3 expression was significantly upregulated in CRC tissues and associated with poor overall survival in both the TCGA and GEO cohorts. High PLXNA3 expression correlated with advanced tumor stage and metastasis. PLXNA3 expression was positively correlated with the infiltration levels of CD4 + T cells, macrophages, neutrophils, and dendritic cells. Functional enrichment analysis suggested potential involvement in glucose metabolism and axonal guidance pathways. In vitro experiments confirmed that PLXNA3 is highly expressed in CRC cell lines and that its knockdown significantly suppresses cell proliferation and migration.

**Conclusion:**

In this study, experimental evidence is provided regarding the promotion of proliferation and migration of CRC by PLXNA3, complementing existing computational predictions. These findings support the role of PLXNA3 as a functionally validated oncogene in CRC and as a candidate for further therapeutic investigation.

## Introduction

Colorectal cancer (CRC) remains among the most common malignancies worldwide, ranking third in incidence and second in cancer-related mortality [1]. Although the incidence of gastric and esophageal cancers has declined in China, the incidence of CRC continues to increase, posing an increasing public health burden [2]. The progression from precancerous lesions to invasive carcinoma typically spans 5 to 10 years, providing a window for early intervention [3]. While the 5-year survival rate for patients with early-stage CRC exceeds 90%, it decreases to less than 10% for patients diagnosed with advanced-stage disease [4]. Early diagnosis and treatment have been demonstrated to significantly reduce CRC-related mortality [5, 6]. Consequently, the identification of novel prognostic biomarkers and therapeutic targets remains an urgent clinical need.

The Cancer Genome Atlas (TCGA) and other public repositories provide comprehensive genomic and clinical data that enable the systematic exploration of cancer-associated genes [7]. Among these genes, genes involved in immune regulation have garnered particular attention given the success of immunotherapy in a subset of CRC patients [8]. Plexins (PLXNs) are a family of transmembrane receptors for class 3 semaphorins that play critical roles in cytoskeletal remodeling, apoptosis, and immune modulation [9]. Plexin A3 (PLXNA3) has been studied primarily in the context of nervous system development, where it contributes to axon guidance [10, 11] and axonal pruning [12] and has been linked to gonadotropin-releasing hormone (GnRH) deficiency [13, 14] and multiple sclerosis [15]. Emerging evidence has also suggested its involvement in breast cancer progression [16, 17], but its role in CRC remains poorly understood.

Recent advances in computational biology have facilitated the identification of prognostic genes related to CRC. A study by Lyu et al. [18] employed machine learning approaches to identify PLXNA3 as an immune-related risk gene in patients with CRC, providing valuable computational insights into its potential prognostic value. However, machine learning predictions, while powerful for biomarker discovery, do not establish causality or reveal the biological functions of candidate genes. The direct effects of PLXNA3 on CRC cell behaviors such as proliferation and migration, as well as its functional relationship with immune infiltration, have not been experimentally validated. This gap limits our understanding of whether PLXNA3 is merely a correlative marker or a functionally active driver of CRC progression.

To address this gap, bioinformatics analysis is combined with in vitro experiments in the present study to comprehensively evaluate the role of PLXNA3 in CRC. We first analyzed PLXNA3 expression and its prognostic value in the TCGA and GEO cohorts. We then investigated its correlation with immune infiltration and performed functional enrichment analysis to generate hypotheses regarding potential pathways. Finally, we conducted in vitro experiments to directly test the hypothesis that PLXNA3 promotes CRC cell proliferation and migration. By providing experimental validation for the oncogenic role of PLXNA3 in CRC, the findings of this study complement existing computational findings and provide a foundation for future mechanistic and therapeutic investigations.

## Materials and methods

### Data acquisition and processing

RNA sequencing data and corresponding clinical information of patients with colon adenocarcinoma (COAD) and rectal adenocarcinoma (READ) (Table 1)were downloaded from The Cancer Genome Atlas (TCGA) repository (https://portal.gdc.cancer.gov/) on February 24, 2022. Gene expression data were log₂-transformed and normalized using the TMM method implemented in the R package “edgeR” (version 3.32.1). Two independent Gene Expression Omnibus (GEO) datasets, GSE41258 and GSE110225, were used for validation of expression and survival. Information regarding immune-related genes was obtained from the ImmPort database (https://www.immport.org/; accessed March 2022).

Differential expression analysis between tumor and normal samples was performed using the limma R package (version 3.46.0) with thresholds of |log₂FC| > 1 and a false discovery rate (FDR) < 0.05. For survival analysis, patients were stratified into high- and low-expression groups based on the median PLXNA3 expression level, as no predefined clinical threshold for PLXNA3 has been established. Univariate and multivariate Cox proportional hazards regression analyses were performed using the R package “survival” (version 3.2–13) to assess the prognostic significance of PLXNA3 and other clinical variables. Genes present at the intersection between immune-related genes and prognosis-related genes (*p* < 0.05 in univariate Cox analysis) were considered candidate immune-related prognostic genes.

### Immune infiltration analysis

The relationship between PLXNA3 expression and immune cell infiltration in CRC was assessed using TIMER2.0 (http://timer.cistrome.org/). Correlation coefficients and statistical significance were calculated for six immune cell types: B cells, CD4 + T cells, CD8 + T cells, macrophages, neutrophils, and dendritic cells.

### Functional enrichment analysis

Genes significantly correlated with PLXNA3 in patients with CRC were identified using the “Similar Gene Detection” module of GEPIA2 (http://gepia2.cancer-pku.cn/), with a Pearson correlation coefficient > 0.3 and *p* < 0.05 as the cutoff. Gene Ontology (GO) and Kyoto Encyclopedia of Genes and Genomes (KEGG) enrichment analyses were performed using the clusterProfiler R package (version 3.18.1). Enriched terms with FDRs < 0.05 were considered to be significantly enriched. Additionally, the functional relevance of PLXNA3 in CRC was explored via CancerSEA (http://biocc.hrbmu.edu.cn/CancerSEA/), which provides single-cell-level functional state annotations.

### Cell culture

Normal human colonic epithelial cells (NCM460) and CRC cell lines (HCT8, LOVO, HT29, and HCT116) were obtained from the American Type Culture Collection (ATCC; Manassas, VA, USA). Cells were cultured in RPMI-1640 or DMEM (Gibco, Thermo Fisher Scientific, Waltham, MA, USA) supplemented with 10% fetal bovine serum (FBS; Gibco) and 1% penicillin‒streptomycin (Gibco) at 37 °C in a humidified atmosphere containing 5% CO₂.

### Small interfering RNA transfection

Small interfering RNAs (siRNAs) targeting PLXNA3 were synthesized by RiBO Biotechnology (Guangzhou, China). HCT8 and LOVO cells were seeded in 6-well plates at a density of 2 × 10⁵ cells per well and transfected with 50 nM si-NC (negative control), si-PLXNA3#1, or si-PLXNA3#2 using Lipofectamine 3000 (Thermo Fisher Scientific, Shanghai, China) according to the manufacturer’s instructions. The culture medium was replaced after 12 h, and the cells were harvested 48 h post-transfection for further analysis. The siRNA sequences used were as follows:

si-PLXNA3-1 sequence: CGAGCACCCTGATGAGTTT.

si-PLXNA3-2 sequence: GAGCTGTATTTCTATGTCA.

si-NC sequence: TTCTCCGAACGTGTCACGT.

The specificity of the siRNA sequence (si‑PLXNA3#2) was verified by BLAST against the human RefSeq RNA database, and no significant homology to off‑target genes was detected.

### Quantitative real-time PCR

Total RNA was extracted from cultured cells using TRIzol reagent (Ambion, Thermo Fisher Scientific) according to the manufacturer’s protocol. RNA concentration and purity were assessed using a NanoDrop spectrophotometer. Reverse transcription was performed using HiScript III RT SuperMix (Vazyme, Nanjing, China). Quantitative real-time PCR (qRT‒PCR) was performed using SYBR Green Mix (Vazyme) on a QuantStudio 5 Real-Time PCR System (Applied Biosystems, Thermo Fisher Scientific). Relative gene expression was calculated using the 2^-ΔΔCt^ method, with GAPDH used as the internal control. All reactions were performed in triplicate. Primer sequences used were as follows:

PLXNA3 forward: 5′-TTTTCCGTGGTCTGGGATGG-3′.

PLXNA3 reverse: 5′-ACAGTTGAAGCGGGGATCAG-3′.

GAPDH forward: 5′-CCTTCCGTGTCCCCACT-3′.

GAPDH reverse: 5′-GCCTGCTTCACCACCTTC-3′.

### Cell proliferation assay

Cell proliferation was assessed using a Cell Counting Kit-8 (CCK-8; Abbkine, Wuhan, China). Briefly, transfected cells were seeded in 96-well plates at a density of 2 × 10³ cells per well. At 0, 24, 48, 72, and 96 h postseeding, 10 µL of CCK-8 solution was added to each well, and the plates were incubated at 37 °C for 2 h. Absorbance at 450 nm was measured using a microplate reader. Each time point was assayed in triplicate, and the experiments were repeated three times independently.

### Colony formation assay

Transfected cells were seeded in 6-well plates at a density of 500 cells per well and cultured for 10–14 days. Colonies were fixed with 4% paraformaldehyde for 15 min, stained with 0.1% crystal violet for 20 min at room temperature, and then washed with PBS. Colonies containing more than 50 cells were counted under an inverted microscope. The experiments were performed in triplicate.

### Transwell migration assay

Cell migration was assessed using Transwell inserts with 8 μm pore sizes (Corning, NY, USA). Transfected CRC cells were harvested, resuspended in serum-free medium, and seeded into the upper chambers at a density of 1 × 10⁵ cells per well. The lower chambers were filled with medium containing 10% FBS as a chemoattractant. After 24 h of incubation at 37 °C, the cells remaining on the upper surface of the membrane were removed with a cotton swab. The migrated cells on the lower surface were fixed with 4% paraformaldehyde, stained with 0.1% crystal violet, and counted in five randomly selected fields under an inverted microscope. Each experiment was performed in triplicate.

### Transwell invasion assay

Cell invasion was assessed using Matrigel-coated Transwell inserts with 8 μm pore size (Corning, NY, USA). The Matrigel (Corning) was diluted 1:4 in serum-free medium, and 50 µL of the diluted Matrigel was added to each upper chamber, followed by incubation at 37 °C for 2 h to allow solidification. Transfected CRC cells were harvested, resuspended in serum-free medium, and seeded into the upper chambers at a density of 1 × 10⁵ cells per well. The lower chambers were filled with medium containing 10% fetal bovine serum (FBS) as a chemoattractant. After 48 h of incubation at 37 °C, the cells remaining on the upper surface of the membrane were removed with a cotton swab. The invaded cells on the lower surface were fixed with 4% paraformaldehyde, stained with 0.1% crystal violet, and counted in five randomly selected fields under an inverted microscope. Each experiment was performed in triplicate with three independent biological replicates.

### Statistical analyses

All the statistical analyses were performed using GraphPad Prism 9.4.0 (GraphPad Software, San Diego, CA, USA) and R software (version 4.1.0). For in vitro experiments, the data are presented as the means ± standard deviations (SDs) from at least three independent experiments. Comparisons between two groups were conducted using unpaired two-tailed Student’s t tests for normally distributed data or Mann‒Whitney U tests for nonnormally distributed data. Comparisons among multiple groups were performed using one-way analysis of variance (ANOVA) followed by Tukey’s post hoc test.

For bioinformatics analyses, survival curves were generated using the Kaplan‒Meier method and compared using the log-rank test. Univariate and multivariate Cox regression analyses were performed to identify independent prognostic factors. For multiple testing in enrichment analyses, p values were adjusted using the Benjamini‒Hochberg method, and an FDR < 0.05 was considered to indicate statistical significance. A two-sided *p* < 0.05 was considered to indicate statistical significance for all the other analyses, unless otherwise specified.

## Results

### PLXNA3 is overexpressed in CRC tissues

**Fig. 1:**
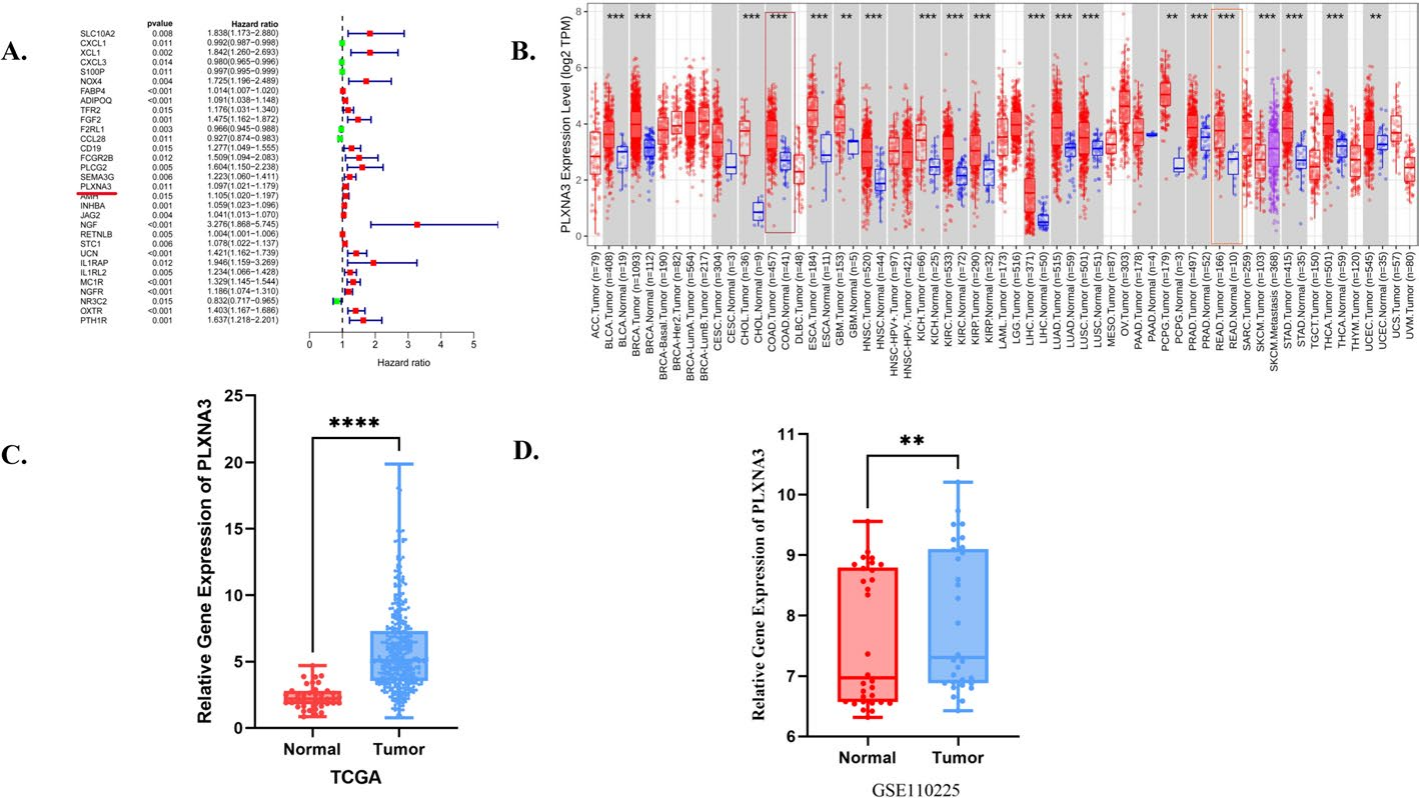
**A** Identification of immune-related genes with significant prognostic value in CRC through univariate Cox proportional hazards analysis. **B** Expression of PLXNA3 in various tumors. Boxplots of PLXNA3 expression in all normal and cancer samples in **C** TCGA cohort and **D** GEO cohort. * *p* < 0.05, **p < 0.01, ***p < 0.001, **** *p* < 0.0001

To identify immune-related genes with prognostic potential in CRC tissues, we first performed univariate Cox regression analysis on immune-related genes obtained from the ImmPort database. This analysis revealed 31 genes significantly associated with CRC prognosis, including 25 with a hazard ratio (HR) > 1 (risk genes) and 6 with an HR < 1 (protective genes) (Fig. 1A). Among these genes, PLXNA3 was identified as a high-risk gene (HR > 1) and was selected for further investigation (Table 1).

Using TIMER2.0, we examined PLXNA3 expression across multiple cancer types. PLXNA3 expression was significantly elevated in various tumors, including colon adenocarcinoma (COAD) and rectal adenocarcinoma (READ), compared with their corresponding normal tissues (Fig. 1B). Consistently, analysis of the TCGA data revealed that PLXNA3 expression was significantly higher in CRC tumor tissues than in adjacent normal tissues (*p* < 0.0001; Fig. 1C). These findings were validated in an independent GEO dataset (GSE110225), in which PLXNA3 expression was also significantly upregulated in CRC samples (*p* < 0.01; Fig. 1D).

### High PLXNA3 expression predicts poor prognosis in CRC

**Fig. 2:**
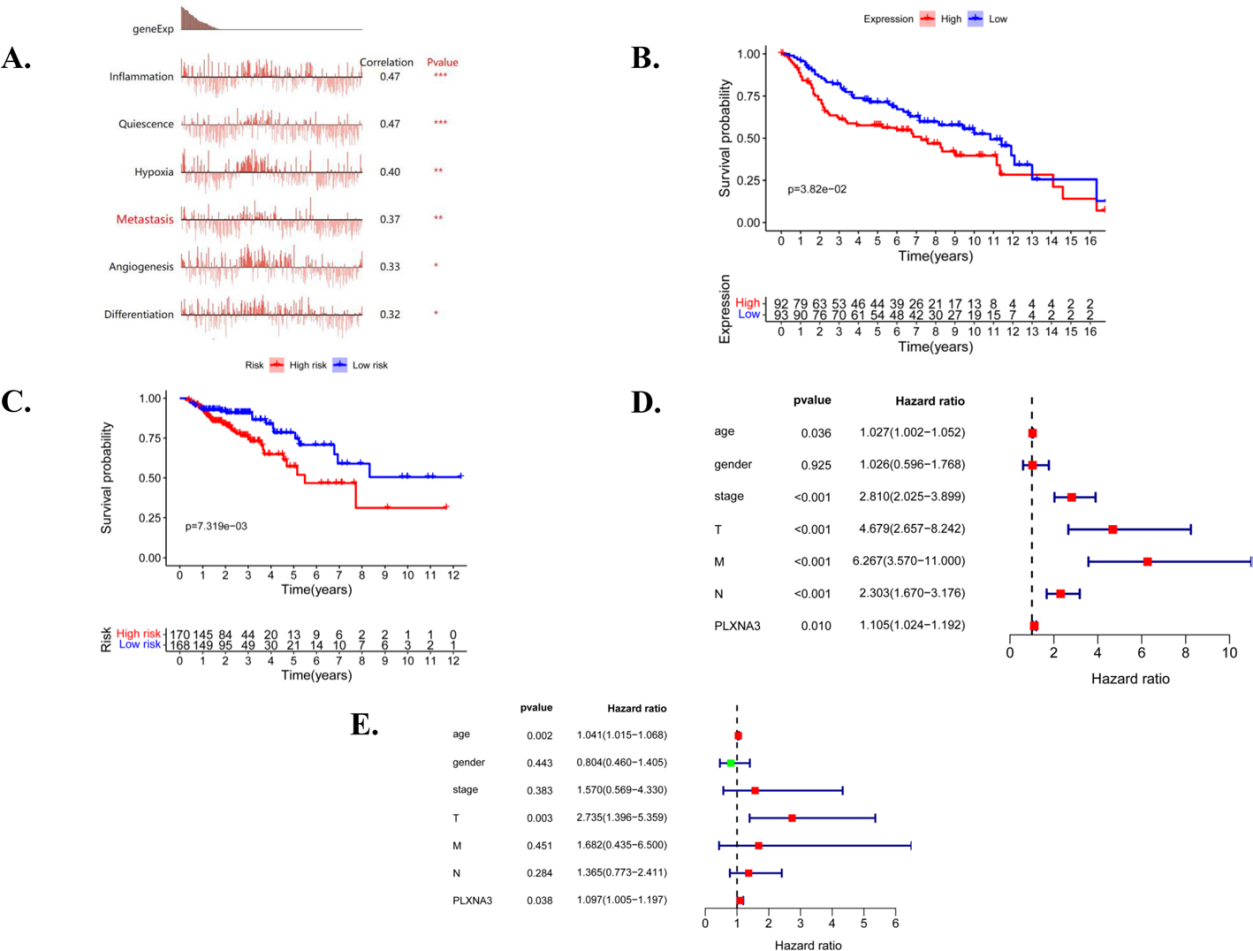
**A** Functional status of PLXNA3 in CRC. Kaplan–Meier survival for low-risk group and high-risk group in **B** TCGA cohort and **C** GEO cohort. **D**, **E** Univariate and multivariate cox regression analyses revealed the PLXNA3 was the independent prognostic factor of CRC in TCGA cohort

We next investigated the functional relevance of PLXNA3 in CRC using the CancerSEA database, which provides single-cell-level functional state annotations. PLXNA3 expression was positively correlated with several cancer-related functional states, including inflammation, metastasis, angiogenesis, and hypoxia (Fig. 2A), suggesting a potential role in tumor progression.

Kaplan-Meier survival analysis of the TCGA data revealed that patients with high PLXNA3 expression had significantly worse overall survival than those with low PLXNA3 expression (*p* < 0.01; Fig. 2B). This prognostic value was validated in the GSE41258 cohort (*p* < 0.05; Fig. 2C). Univariate Cox regression analysis revealed that PLXNA3 expression, along with age, T stage, N stage, M stage, and clinical stage, was significantly associated with overall survival (Fig. 2D). Importantly, multivariate Cox regression analysis confirmed that PLXNA3 expression remained an independent prognostic factor for CRC after adjusting for other clinical variables (HR = 1.097; 95% CI: 1.005–1.197; *p* = 0.038; Fig. 2E; Table 2).

**Table 1:** The baseline characteristics of CRC patients in TCGA datasets

Clinical characteristics	TCGA cohort (*n* = 385)
Age (median, range)	68.5 (31–90)
Vital status	
Dead	79
Alive	306
Gender	
Female	180
Male	205
T Stage	
T1	9
T2	68
T3	263
T4	44
Tis	1
N Stage	
N0	231
N1	88
N2	66
N3	0
M Stage	
M0	286
M1	54
Mx	39
Unknow	6
Clinical Stage	
Stage I	66
Stage II	151
Stage III	103
Stage IV	54
Unknown	11

**Table 2:** The multivariate Cox regression analysis of TCGA

	HR	HR.95 L	HR.95 H	p value
Age	1.041063	1.014943	1.067856	0.001909
Gender	0.803568	0.459518	1.405216	0.443117
Stage	1.570218	0.569397	4.330167	0.383309
T	2.735441	1.39622	5.359213	0.003361
M	1.68246	0.435478	6.500151	0.450581
N	1.36492	0.772711	2.411	0.283857
PLXNA3	1.09684	1.005259	1.196765	0.037722

HR: hazard ratio

### PLXNA3 expression is correlated with advanced clinicopathological features

**Fig. 3:**
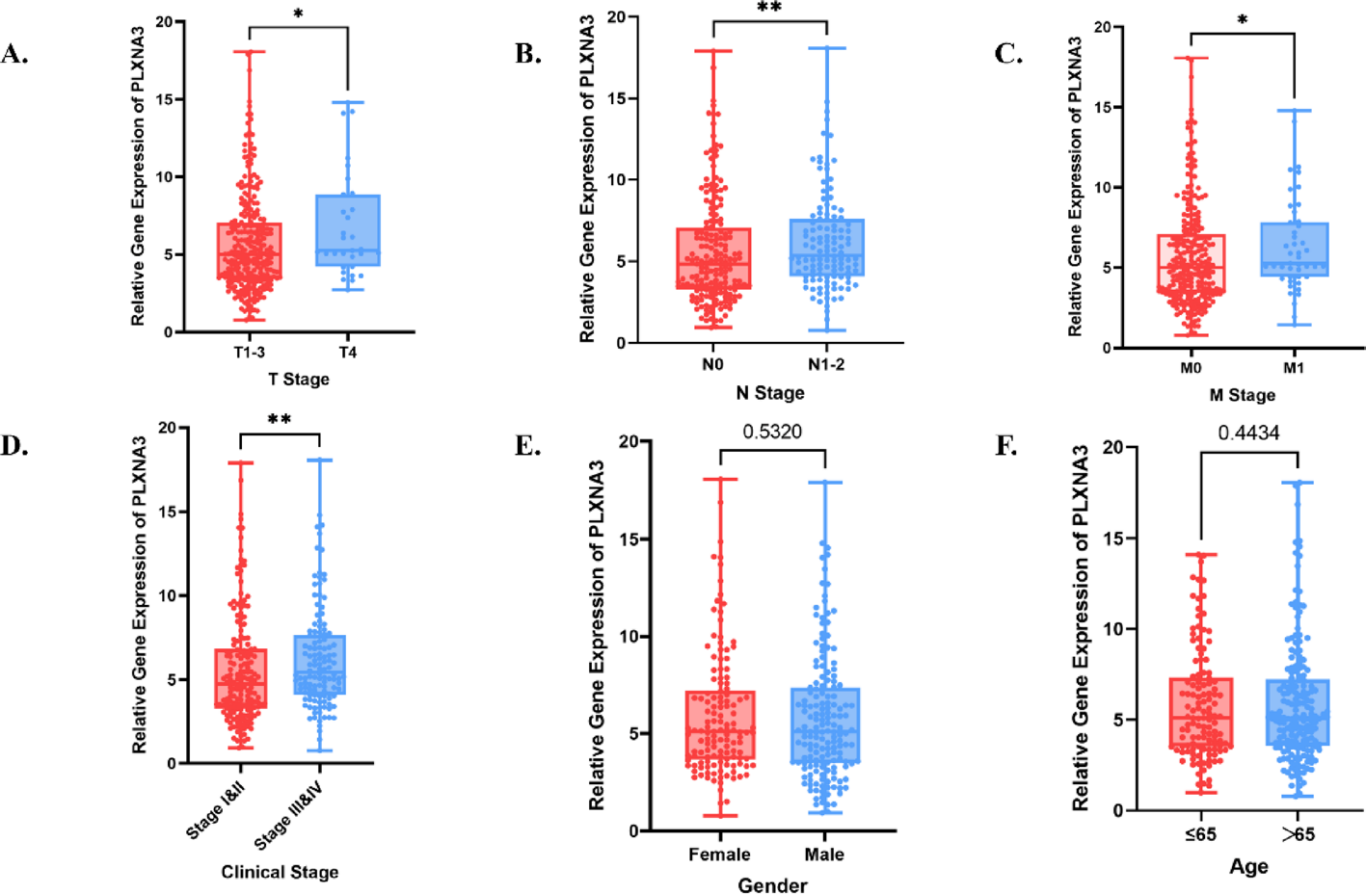
Association between PLXNA3 expression and clinicopathological characteristics in CRC. Boxplots showing the relationship between PLXNA3 expression and (A) T stage, (B) N stage, (C) M stage, (D) clinical stage, (E) gender, and (F) age in the TCGA cohort. *p < 0.05, **p < 0.01.

To further explore the clinical significance of PLXNA3, we analyzed its association with clinicopathological parameters in the TCGA cohort. High PLXNA3 expression was significantly associated with advanced T stage (*p* < 0.05), lymph node metastasis (N stage, *p* < 0.01), distant metastasis (M stage, *p* < 0.05), and overall clinical stage (*p* < 0.01) (Fig. 3). These associations suggest that PLXNA3 may be involved in CRC progression and metastasis.

### PLXNA3 is associated with immune infiltration and metabolic pathways

**Fig. 4:**
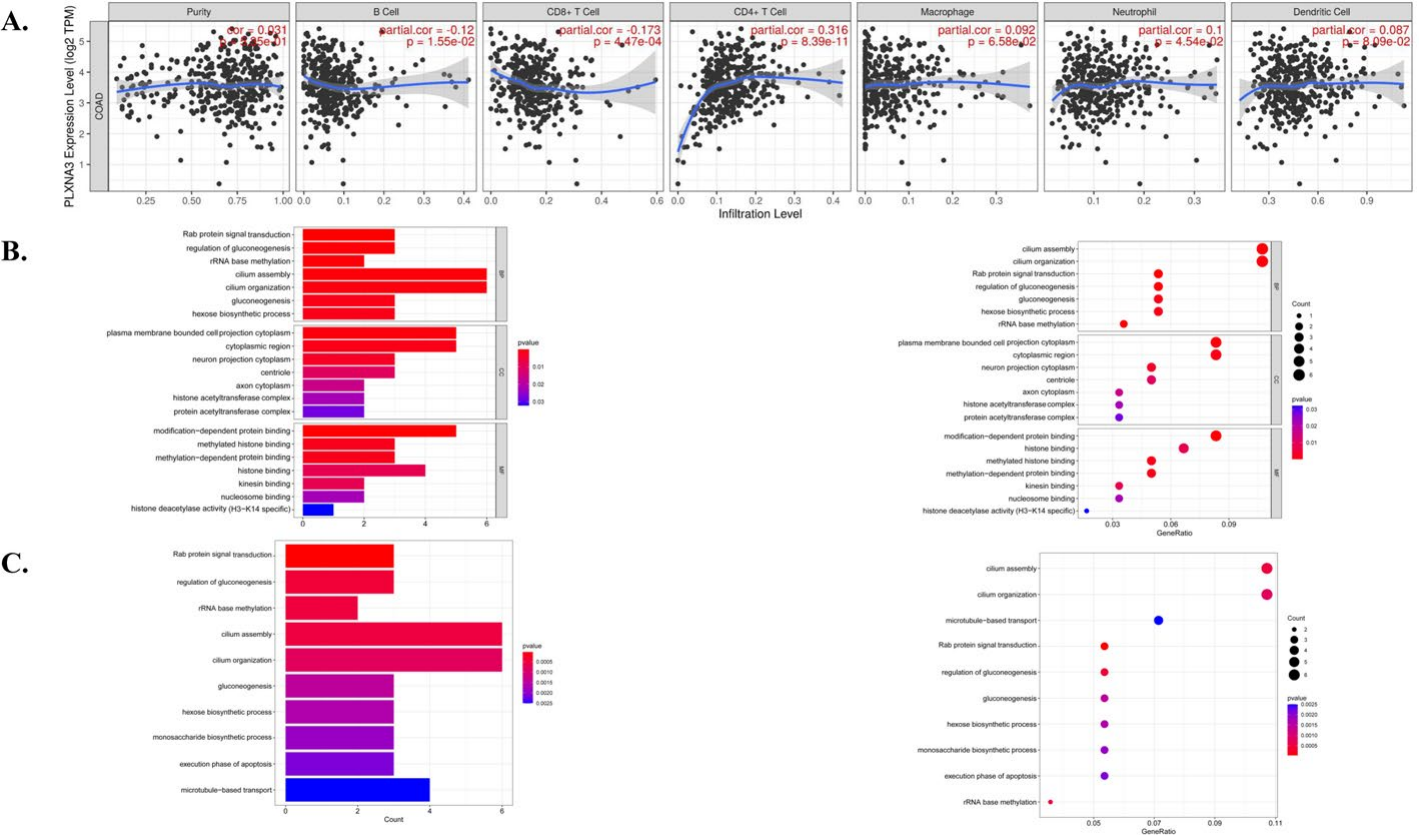
**A** The relationship of PLXNA3 expression with immune infiltration level in CRC. Functional enrichment analysis depending on “similar” modules of GEPIA database. **B** Bar plot and Bubble chart of Gene Ontology enrichment analysis. **C** Bar plot and Bubble chart of KEGG enrichment analysis

Given the role of plexins in immune regulation, we assessed the correlation between PLXNA3 expression and immune cell infiltration using TIMER2.0. PLXNA3 expression was positively correlated with the infiltration levels of CD4 + T cells (*R* = 0.341; *p* < 0.001), macrophages (*R* = 0.389; *p* < 0.001), neutrophils (*R* = 0.402; *p* < 0.001), and dendritic cells (*R* = 0.378; *p* < 0.001) in CRC (Fig. 4A). No significant correlation was observed with CD8 + T cells or B cells.

To generate hypotheses about potential underlying mechanisms, we performed GO and KEGG enrichment analyses on genes significantly correlated with PLXNA3 expression in CRC. GO analysis revealed enrichment in biological processes related to glucose metabolism, including gluconeogenesis, the hexose biosynthetic process, and the monosaccharide metabolic process (Fig. 4B). KEGG pathway analysis revealed enrichment in pathways such as “axonal guidance,” “central carbon metabolism in cancer,” and “glucagon signaling pathway” (Fig. 4C). These enrichment analyses results allow the development of hypotheses related to potential downstream pathways, although direct experimental validation of these correlations is needed in future studies.

### Validation of PLXNA3 expression in CRC cell lines

**Fig. 5:**
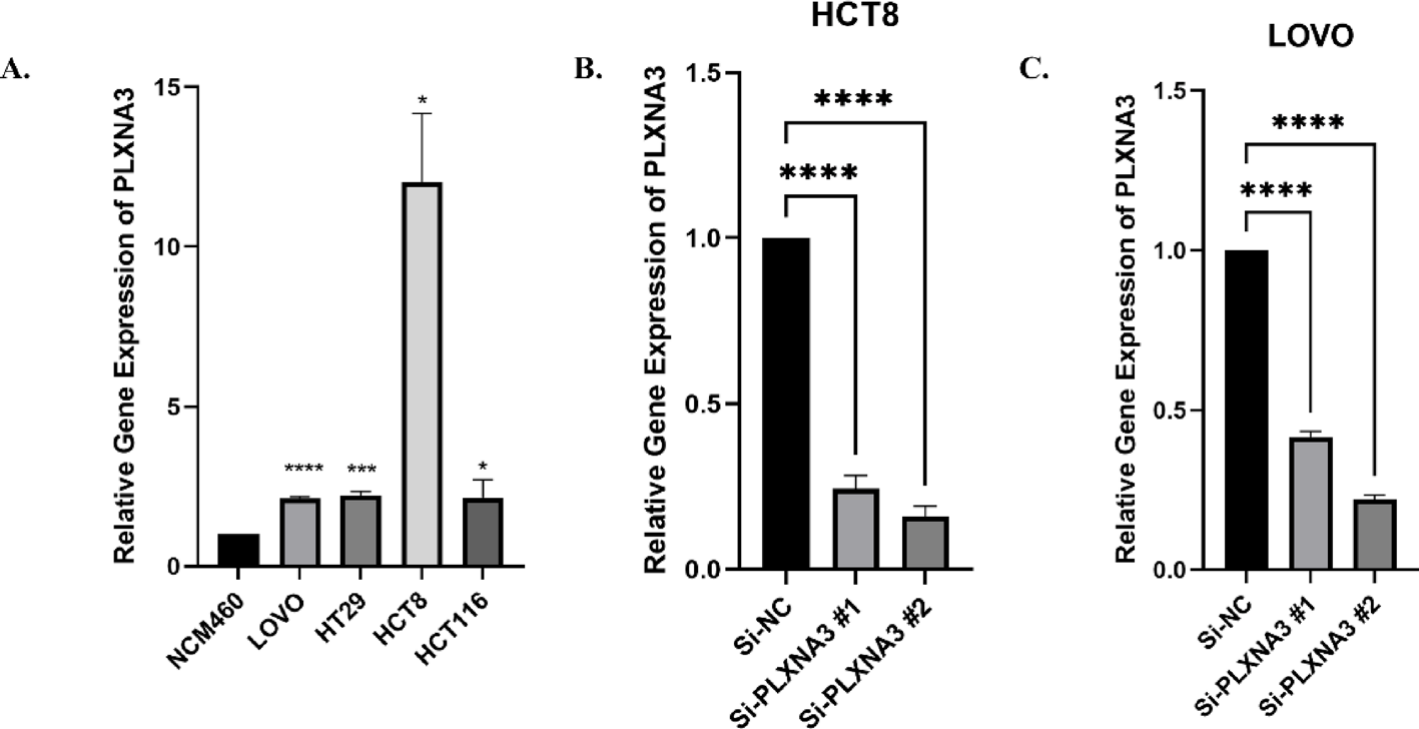
**A** The mRNA expression levels of PLXNA3 in four CRC cell lines (LOVO, HT29, HCT8, HCT116). **B**, **C** Detection of PLXNA3 knockdown by RT-qPCR. Mean ± SD (*p < 0.05, **p < 0.01, ***p < 0.001, ****p < 0.0001)

To experimentally validate the bioinformatics findings, we first examined PLXNA3 mRNA expression in a panel of CRC cell lines (HCT8, LOVO, HT29, and HCT116) and normal colonic epithelial cells (NCM460) using qRT‒PCR. Consistent with the tissue-level data, PLXNA3 expression was significantly higher in all four CRC cell lines than in NCM460 cells (Fig. 5A). Among these cells, HCT8 and LOVO cells presented the highest PLXNA3 expression and were therefore selected for subsequent loss-of-function experiments.

To knock down PLXNA3 expression, we transfected HCT8 and LOVO cells with two independent siRNAs targeting PLXNA3 (si-PLXNA3#1 and si-PLXNA3#2). qRT‒PCR analysis confirmed that compared with negative control siRNA (si-NC), both siRNAs significantly decreased PLXNA3 mRNA levels (Fig. 5B, C). si-PLXNA3#2 resulted in slightly increased knockdown efficiency and was used for all subsequent functional assays.

### PLXNA3 knockdown inhibits CRC cell proliferation and migration

**Fig. 6:**
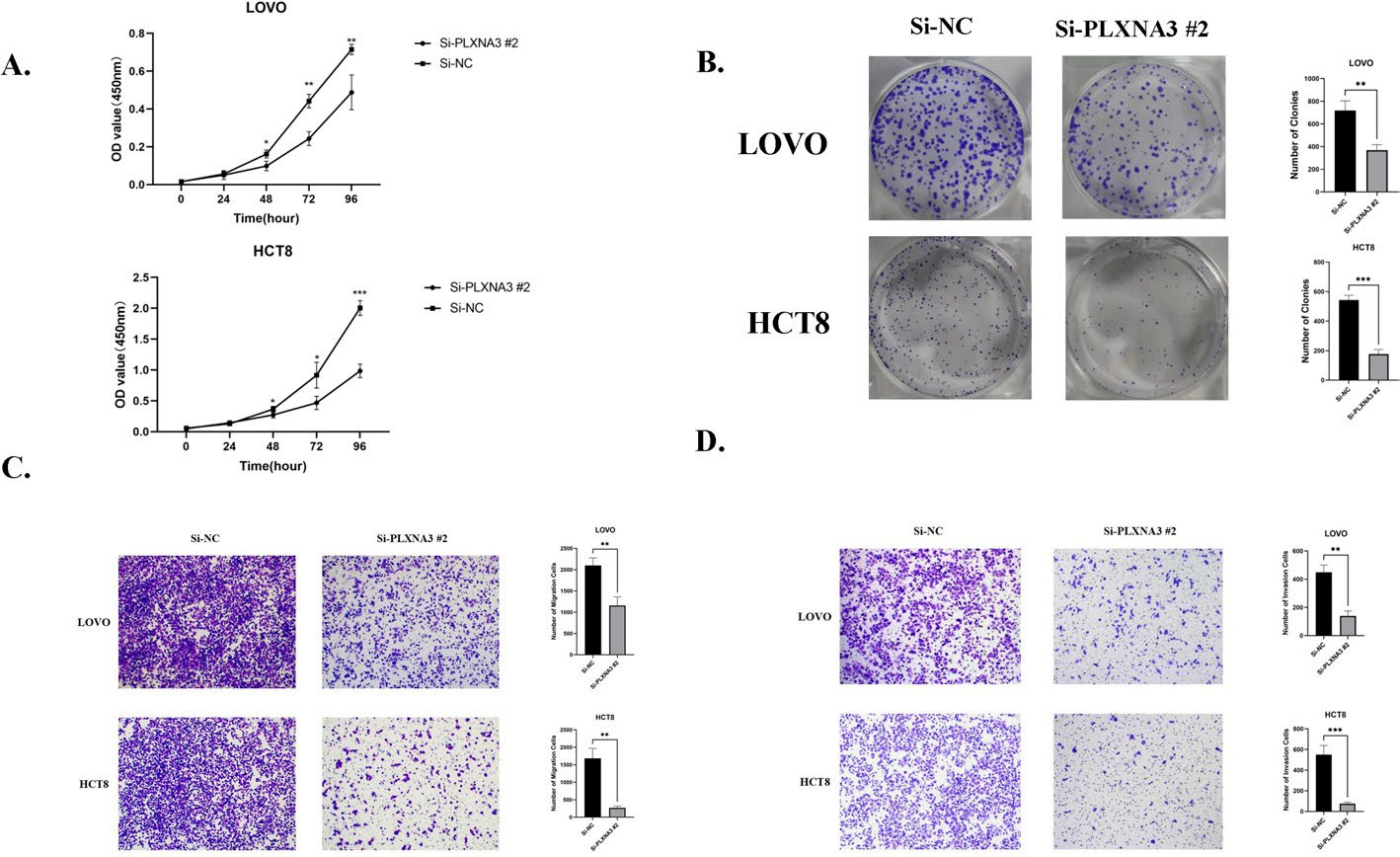
The effects of PLXNA3 silencing on proliferation were evaluated with **A** CCK-8 assay and **B** Clone formation assay. **C** The effects of PLXNA3 silencing on migration were evaluated by Transwell assay. **D** The effects of PLXNA3 silencing on invasion were evaluated by Matrigel-coated Transwell invasion assay.Mean ± SD (*p < 0.05, **p < 0.01, ***p < 0.001)

To assess the functional role of PLXNA3 in CRC, we evaluated the effects of PLXNA3 knockdown on cell proliferation and migration. CCK-8 assays revealed that PLXNA3 knockdown significantly reduced the viability of both HCT8 and LOVO cells at 48, 72, and 96 h post-transfection, whereas no differences were observed at 0–24 h (Fig. 6A). Consistently, colony formation assays revealed that PLXNA3 knockdown significantly decreased the number and size of colonies formed by both cell lines (Fig. 6B), indicating reduced proliferative capacity.

We next examined the effect of PLXNA3 knockdown on cell migration using Transwell assays. As shown in Fig. 6C, compared with control groups, PLXNA3 knockdown significantly reduced the number of migrated HCT8 and LOVO cells. Taken together, these results reveal that PLXNA3 promotes CRC cell proliferation and migration in vitro.

### PLXNA3 knockdown suppresses CRC cell invasion

To further evaluate the role of PLXNA3 in CRC progression, Matrigel-coated Transwell invasion assays were performed. As shown in Fig. 6D, PLXNA3 knockdown significantly reduced the invasive capacity of both HCT8 and LOVO cells compared with the negative control (*p* < 0.01). These findings indicate that PLXNA3 promotes not only migration but also invasion of CRC cells in vitro.

## Discussion

In this study, we systematically evaluated the expression, prognostic significance, and functional role of PLXNA3 in CRC through a combination of bioinformatics analysis and in vitro experiments. Our findings reveal that PLXNA3 expression is significantly upregulated in CRC tissues and is correlated with poor prognosis and advanced clinicopathological features. More importantly, we provide the first experimental evidence that PLXNA3 knockdown suppresses CRC cell proliferation and migration, directly demonstrating its oncogenic function in this malignancy. These results support previous observations in patients with breast cancer [16, 17] and establish PLXNA3 as a functional player in CRC progression.

The association between PLXNA3 expression and immune infiltration, particularly its positive correlations with CD4 + T cells, macrophages, neutrophils, and dendritic cells, suggests a potential association between PLXNA3 expression and the tumor immune microenvironment. These findings align with the emerging concept of neuroimmune crosstalk[19, 20], in which semaphorin-plexin signaling serves as a molecular bridge between the nervous system and the immune system [21–23]. Plexins regulate immune cell migration and function [22], and our results suggest that the expression of PLXNA3 in tumor cells might influence immune cell recruitment or activation. However, the correlative nature of these data should be emphasized: whether PLXNA3 expression is functionally linked to immune infiltration or whether its expression is simply coregulated with immune-related genes remains to be experimentally determined.

Functional enrichment analysis suggested potential links between PLXNA3 and glucose metabolism as well as axonal guidance pathways. The association with glucose metabolism is particularly intriguing given the well-established role of metabolic reprogramming during CRC progression [24–28]. Several recent studies have highlighted the importance of glycolysis and other metabolic pathways in CRC [25–28], and our findings position PLXNA3 as a potential modulator of these processes. Similarly, the enrichment of axonal guidance pathways is consistent with the established role of PLXNA3 in nervous system development [10, 12–14] and may reflect broader connections between neuronal signaling molecules and cancer biology. It is important to emphasize, however, that these enrichment results are hypothesis generating rather than confirmatory. Direct experimental validation—for example, metabolomic profiling or pathway-specific functional assays—is needed to determine whether PLXNA3 truly regulates glucose metabolism in CRC cells.

### Contextualization within recent literature

During the preparation of this manuscript, a study by Lyu et al. [18] employed machine learning and multiomics approaches to identify PLXNA3 as an immune-related risk gene in CRC. Their computational analysis, which included LASSO regression and random forest modeling, revealed that PLXNA3 is part of a prognostic signature and demonstrated its correlation with immune infiltration. Our bioinformatics findings are largely consistent with their results, providing independent validation of the prognostic value and immune associations of PLXNA3. Extending their work, our study provides direct experimental evidence that PLXNA3 knockdown suppresses CRC cell proliferation and migration, thereby transforming a computationally predicted risk gene into a functionally validated oncogene. This convergence of bioinformatics prediction and experimental validation strengthens the biological plausibility of PLXNA3 as a driver of CRC progression.

## Limitations

This study has several limitations that should be acknowledged. First, while we designed two independent siRNAs to minimize off-target effects, functional assays were performed primarily using a single siRNA (si-PLXNA3#2) because of its higher knockdown efficiency. Validation with the second siRNA would strengthen the conclusions. Future studies should include validation using both independent siRNAs to definitively exclude off-target effects. Second, the functional link between PLXNA3 and glucose metabolism, while suggested by enrichment analysis, remains speculative and requires direct experimental validation through metabolomic profiling or metabolic flux assays. Third, although we demonstrated that PLXNA3 knockdown inhibits CRC cell proliferation and migration, the underlying molecular mechanisms—such as downstream signaling pathways or interacting partners—have not been elucidated. Fourth, all bioinformatics analyses were based on public databases with inherent biases, including potential batch effects and differences in sequencing platforms. Fifth, the immune infiltration correlations, while statistically significant, are correlative and do not establish causality; functional studies using coculture systems or in vivo models are needed to determine whether PLXNA3 directly influences immune cell recruitment. Sixth, the use of median expression as a cutoff for patient stratification, while commonly employed, may introduce bias; more robust methods such as X-tile analysis could be applied in future studies. Seventh, although Matrigel-coated Transwell invasion assays have now been performed and demonstrate that PLXNA3 knockdown suppresses CRC cell invasion (Fig. 6D), the molecular mechanisms through which PLXNA3 regulates invasion have not been elucidated and warrant further investigation.Eighth, our in vitro findings, though robust, need to be confirmed in in vivo models to assess the tumorigenic potential of PLXNA3 in a physiological context. Ninth, the clinical utility of PLXNA3 as a prognostic biomarker requires validation in independent, prospective cohorts with standardized treatment protocols.

## Future directions and conclusions

Despite these limitations, this study makes a significant contribution by providing the first experimental validation of the oncogenic role of PLXNA3 in CRC. Future studies should focus on (1) identifying the molecular mechanisms by which PLXNA3 promotes proliferation and migration, including potential downstream effectors such as Rho GTPases or cytoskeletal regulators; (2) investigating its potential role in metabolic reprogramming through metabolomic and functional analyses; (3) exploring its functional impact on immune cell recruitment and activation using coculture systems and in vivo models; and (4) evaluating its therapeutic potential in preclinical animal models, such as xenograft or orthotopic tumor models.

## Conclusion

In conclusion, the findings of this study revealed that PLXNA3 expression is significantly upregulated in colorectal cancer tissues and is correlated with poor prognosis. Experimental validation confirmed that PLXNA3 knockdown suppresses CRC cell proliferation and migration, providing the first functional evidence for its oncogenic role in patients with this malignancy. While its association with immune infiltration and metabolic pathways warrants further investigation, the current findings establish PLXNA3 as a functionally validated oncogene in CRC and support its continued evaluation as a potential biomarker and therapeutic target.

## Data Availability

The datasets generated and analyzed during the current study are available from public repositories. The Cancer Genome Atlas (TCGA) data for colon adenocarcinoma (COAD) and rectal adenocarcinoma (READ) are accessible at: [https://portal.gdc.cancer.gov/projects/TCGA-COAD] (https:/portal.gdc.cancer.gov/projects/TCGA-COAD) and https://portal.gdc.cancer.gov/projects/TCGA-READ. The Gene Expression Omnibus (GEO) datasets used in this study are available under accession numbers GSE41258 (https://www.ncbi.nlm.nih.gov/geo/query/acc.cgi?acc=GSE41258) and GSE110225 (https://www.ncbi.nlm.nih.gov/geo/query/acc.cgi?acc=GSE110225). The analysis code is available on GitHub at https://github.com/fjh9947/aabbcc.git.

## Code availability

The analysis code is available on GitHub at https://github.com/fjh9947/aabbcc.git.

## Abbreviations

CRC: Colorectal cancer
PLXNA3: Plexin A3
TCGA: The Cancer Genome Atlas
GEO: Gene Expression Omnibus
TIMER2.0: Tumor Immune Estimation Resource 2.0
GEPIA: Gene Expression Profiling Interactive Analysis
GnRH: Gonadotropin-releasing Hormone
qRT-PCR: Quantitative real-time PCR
KEGG: Kyoto Encyclopedia of Genes and Genomes
GO: Gene Ontology
COAD: Colon adenocarcinoma
